# Correction: Evaluation of an autonomous acoustic surveying technique for grassland bird communities in Nebraska

**DOI:** 10.1371/journal.pone.0322590

**Published:** 2025-04-10

**Authors:** Grace E. Schuster, Leroy J. Walston, Andrew R. Little

The equation in the Recall subsection of the Methods contains an error. Please view the complete, correct equation here:

$\frac{true\;positives}{true\;positives+false\;negatives}$
