# Supplementary material for: Evaluation of an autonomous acoustic surveying technique for grassland bird communities in Nebraska
Source: PLoS One. 2024 Jul 5;19(7):e0306580. doi: 10.1371/journal.pone.0306580 (PMC11226020; doi:10.1371/journal.pone.0306580)
Supplement: S1 File — (DOCX) [file pone.0306580.s001.docx]

**SUPPORTING INFORMATION**

**Title:** Evaluation of an Autonomous Acoustic Surveying Technique for Grassland Bird Communities in Nebraska

**Supplementary Material**

Table of Contents SI-2

S1 Table. Details on the dates, times, and species recorded during the point count surveys SI-3

S2 Table. Summary of BirdNET recall from 50 random 120-s clips SI-4

S3 Table. Summary table of all bird species detected by BirdNET during the study period SI-6

S1 Fig. Acoustic Monitor attached to the top of a polyvinyl chloride (PVC) pole SI-9

S2 Fig. Acoustic recorder installed in the center pivot area of a corn field SI-9

S3 Fig. Acoustic recorder installed in a grassland field SI-10

**S1 Table. Details on the dates, times, and species recorded during the point count surveys.**

| **Site ID** | **Visit No.** | **Date** | **Species Richness** | **Species Codes^1^** |
| --- | --- | --- | --- | --- |
| Site 4 | 1 | 19/05/2022 | 6 | CHSP, DICK, GRSP, PHEA, RWBL, WEME |
| Site 4 | 2 | 14/6/2022 | 7 | BOBW, DICK, EAKI, GRSP, PHEA, RWBL, WEME |
| Site 4 | 3 | 13/07/2022 | 8 | BASP, BOBW, EAKI, GRSP, MODO, PHEA, RWBL, WEME |
| Site 1 | 1 | 25/05/2022 | 7 | CHSP, DICK, GRAC, MODO, PHEA, RWBL, WEME |
| Site 1 | 2 | 14/6/2022 | 6 | DICK, MODO, PHEA, ROBI, RWBL, WEME |
| Site 1 | 3 | 13/07/2022 | 5 | GOLD, GRSP, MODO, TRSW, WEME |
| Site 9 | 1 | 20/05/2022 | 10 | BAOR, BARN, CROW, DICK, GRSP, MODO, PHEA, ROBI, RWBL, WEME |
| Site 9 | 2 | 16/6/2022 | 8 | BOBW, BRTH, DICK, GRSP, HOSP, PHEA, RWBL, WEME |
| Site 9 | 3 | 12/7/2022 | 8 | DICK, EAKI, EUST, GRSP, MODO, PHEA, TURK, WEME |
| Site 7 | 1 | 19/05/2022 | 9 | BLUE, DICK, ECDO, HOWR, PHEA, ROBI, RWBL, TUVU, WEME |
| Site 7 | 2 | 6/6/2022 | 12 | BLUE, CYTH, DICK, ECDO, EUST, FLYC, HOWR, MODO, RHWO, ROBI, RWBL, YEWA |
| Site 7 | 3 | 13/07/2022 | 14 | BANK, BAOR, BEVI, BLUE, GRAC, HOWR, MODO, NOFL, PHEA, RHWO, ROBI, RWBL, TRSW, WEME |
| Site 2 | 1 | 25/05/2022 | 7 | EAKI, ECDO, GRAC, PHEA, ROBI, RWBL, WEME |
| Site 2 | 2 | 14/6/2022 | 7 | COWB, CROW, HOLA, MODO, PHEA, ROBI, RWBL |
| Site 2 | 3 | 19/07/2022 | 5 | ECDO, GRAC, GRSP, HOWR, ROBI |
| Site 8 | 1 | 19/05/2022 | 8 | BARN, BLUE, BOBW, DICK, MODO, ROBI, RWBL, TRSW |
| Site 8 | 2 | 6/6/2022 | 10 | BLUE, CARD, EAKI, G GRACK, GRAC, HOLA, HOWR, ROBI, RWBL, WEME |
| Site 8 | 3 | 18/07/2022 | 4 | MODO, ROBI, RWBL, TUVU |
| Site 10 | 1 | 20/05/2022 | 8 | BOBW, CHSP, CROW, FISP, GRSP, MODO, RWBL, WEME |
| Site 10 | 2 | 16/6/2022 | 2 | HOLA, WEME |
| Site 10 | 3 | 18/07/2022 | 6 | BARN, EAKI, GOLD, GRAC, RWBL, WEME |
| Site 3 | 1 | 19/05/2022 | 7 | DICK, FISP, GRSP, KILL, RWBL, WEKI, WEME |
| Site 3 | 2 | 13/6/2022 | 4 | DICK, EAKI, RWBL, WEME |
| Site 3 | 3 | 13/07/2022 | 5 | HOLA, KILL, MODO, RWBL, WEME |
| Site 12 | 1 | 24/05/2022 | 5 | DICK, GRSP, PHEA, TUVU, WEME |
| Site 12 | 2 | 13/6/2022 | 7 | DICK, EAKI, GRSP, MODO, PHEA, RWBL, WEME |
| Site 12 | 3 | 12/7/2022 | 5 | GOLD, GRSP, HOLA, PHEA, WEME |
| Site 11 | 1 | 25/05/2022 | 7 | GRSP, HOWR, MODO, ORCH, PHEA, RWBL, WEME |
| Site 11 | 2 | 13/6/2022 | 5 | DICK, EAKI, GRSP, PHEA, WEME |
| Site 11 | 3 | 13/07/2022 | 5 | BLGR, DICK, MODO, PHEA, WEME |

^1^ Species Codes: BANK = Bank Swallow, BAOR = Baltimore Oriole, BARN = Barn Swallow, BASP = Baird’s Sparrow, BEVI = Bell’s Vireo, BLGR = Blue Grosbeak, BLUE = Blue Jay, BOBW = Bobwhite, BRTH = Brown Thrasher, CARD = Northern Cardinal, CHSP = Chipping Sparrow, COWB = Brown-headed Cowbird, CROW = American Crow, CYTH = Common Yellowthroat, DICK = Dickcissel, EAKI = Eastern Kingbird, ECDO = Eurasian Collared Dove, EUST = European Starling, FISP - Field Sparrow, FLYC = Great Crested Fly-Catcher, G. GRACK = Great Tailed Grackle, GOLD = American Goldfinch, GRAC = Common Grackle, GRSP = Grasshopper Sparrow, HOLA = Horned Lark, HOSP = House Sparrow, HOWR = House Wren, KILL = Killdeer, MODO = Mourning Dove, NOFL = Northern Flicker, ORCH = Orchard Oriole, PHEA = Ring-necked Pheasant, RHWO = Red-headed Woodpecker, ROBI = American Robin, RWBL = Red-winged Blackbird, TRSW = Tree Swallow, TURK = Wild Turkey, TUVU = Turkey Vulture, WEKI = Western Kingbird, WEME = Western Meadowlark, YEWA = Yellow Warbler.

**S2 Table. Comparison of BirdNET annotation with manual annotation for the 20 focal species based on 50 randomly-generated 120-s acoustic clips.**

| **120-s File Name** | **N Focal Species Detected by BirdNET** | **BirdNET Species Codes** | **N Focal Species Manually Detected** | **Manual Species Codes^1^** |
| --- | --- | --- | --- | --- |
| File-1.wav | 10 | DICK, GRSP, HOLA, MODO, BOBW, RWBL, SASP, TRSW, WEKI, WEME | 4 | MODO, PHEA, RWBL, WEME |
| File-2.wav | 10 | BLGR, GRAC, DICK, GRSP, HOLA, BOBW, RWBL, PHEA, TRSW, WEME | 5 | DICK, MODO, PHEA, RWBL, WEME |
| File-3.wav | 9 | BLGR, DICK, GRSP, HOLA, MODO, BOBW, PHEA, TRSW, WEME | 7 | BOBW, DICK, GRSP, MODO, PHEA, RWBL, WEME |
| File-4.wav | 7 | BLGR, DICK, GRSP, MODO, BOBW, SASP, WEME | 4 | BLGR, GRSP, MODO, ROBI |
| File-5.wav | 1 | BLUE | 1 | NONE |
| File-6.wav | 1 | ROBI | 1 | ROBI |
| File-7.wav | 7 | BLGR, DICK, GRSP, HOLA, BOBW, WEKI, WEME | 2 | HOLA, WEME |
| File-8.wav | 0 | NONE | 0 | NONE |
| File-9.wav | 0 | NONE | 0 | NONE |
| File-10.wav | 7 | BLGR, EAKI, GRSP, MODO, BOBW, SASP, WEME | 3 | BOBW, MODO, WEME |
| File-11.wav | 7 | GRAC, GRSP, HOLA, RWBL, PHEA, SASP, WEME | 3 | PHEA, RWBL, WEME |
| File-12.wav | 9 | DICK, EAKI, GRSP, HOLA, RWBL, PHEA, SASP, WEKI, WEME | 4 | DICK, EAKI, PHEA, RWBL |
| File-13.wav | 10 | BLGR, DICK, EAKI, GRSP, BOBW, RWBL, PHEA, SASP, WEKI, WEME | 5 | DICK, GRSP, PHEA, RWBL, WEME |
| File-14.wav | 0 | NONE | 1 | NONE |
| File-15.wav | 4 | ROBI, BLGR, EAKI, RWBL | 2 | RWBL, SASP |
| File-16.wav | 4 | DICK, GRSP, HOLA, SASP | 1 | HOLA |
| File-17.wav | 7 | BLGR, DICK, GRSP, HOLA, SASP, TRSW, WEME | 1 | HOLA |
| File-18.wav | 10 | GOLD, BLGR, BLUE, DICK, GRSP, HOLA, RWBL, SASP, TRSW, WEME | 3 | HOLA, PHEA, WEME |
| File-19.wav | 3 | BLGR, HOLA, BOBW | 2 | BOBW, HOLA |
| File-20.wav | 2 | GOLD, BLUE | 0 | NONE |
| File-21.wav | 7 | ROBI, GRAC, GRSP, HOLA, MODO, SASP, WEME | 3 | GRAC, ROBI, WEME |
| File-22.wav | 9 | GOLD, ROBI, BLGR, DICK, GRSP, HOLA, BOBW, TRSW, WEME | 4 | DICK, PHEA, ROBI, WEME |
| File-23.wav | 8 | BLGR, DICK, EAKI, GRSP, BOBW, SASP, TRSW, WEME | 3 | BLUE, DICK, WEME |
| File-24.wav | 10 | BLGR, BLUE, GRAC, DICK, EAKI, GRSP, HOLA, BOBW, SASP, WEME | 3 | BLUE, EAKI, ROBI |
| File-25.wav | 3 | GOLD, BLUE, HOLA | 1 | BLUE |
| File-26.wav | 6 | ROBI, GRSP, HOLA, MODO, SASP, WEME | 3 | HOLA, ROBI, WEME |
| File-27.wav | 9 | BLGR, DICK, GRSP, HOLA, BOBW, PHEA, SASP, TRSW, WEME | 3 | HOLA, PHEA, WEME |
| File-28.wav | 2 | ROBI, BLUE | 0 | NONE |
| File-29.wav | 8 | BLGR, DICK, GRSP, MODO, BOBW, PHEA, WEKI, WEME | 6 | BLGR, BOBW, DICK, MODO, PHEA, ROBI |
| File-30.wav | 1 | GRSP | 0 | NONE |
| File-31.wav | 7 | GRSP, HOLA, MODO, RWBL, PHEA, TRSW, WEME | 6 | GRSP, MODO, PHEA, ROBI, RWBL, WEME |
| File-32.wav | 10 | BLGR, DICK, GRSP, HOLA, BOBW, RWBL, PHEA, SASP, TRSW, WEME | 5 | DICK, GRSP, PHEA, RWBL, WEME |
| File-33.wav | 8 | BLGR, CONI, DICK, EAKI, GRSP, BOBW, PHEA, WEME | 6 | CONI, DICK, GRSP, PHEA, RWBL, WEME |
| File-34.wav | 8 | BLGR, DICK, EAKI, GRSP, HOLA, BOBW, SASP, WEME | 2 | BLGR, GRSP |
| File-35.wav | 7 | GOLD, BLGR, HOLA, MODO, BOBW, PHEA, WEME | 5 | BLGR, BOBW, GOLD, PHEA, WEME |
| File-36.wav | 5 | GRAC, GRSP, HOLA, PHEA, WEME | 2 | PHEA, WEME |
| File-37.wav | 7 | DICK, GRSP, HOLA, PHEA, SASP, TRSW, WEME, | 6 | HOLA, PHEA, RWBL, SASP, TURK, WEME |
| File-38.wav | 5 | GRSP, HOLA, BOBW, PHEA, WEME | 6 | BOBW, HOLA, MODO, PHEA, RWBL, WEME |
| File-39.wav | 7 | BLGR, DICK, GRSP, HOLA, BOBW, RWBL, WEME | 1 | RWBL |
| File-40.wav | 0 | NONE | 0 | NONE |
| File-41.wav | 0 | NONE | 5 | DICK, GRSP, MODO, PHEA, WEME |
| File-42.wav | 5 | GRSP, HOLA, PHEA, TRSW, WEME | 6 | DICK, GRSP, HOLA, MODO, PHEA, WEME |
| File-43.wav | 0 | NONE | 5 | DICK, GRSP, MODO, PHEA, WEME |
| File-44.wav | 0 | NONE | 2 | HOLA, WEME |
| File-45.wav | 8 | BLGR, CONI, DICK, GRSP, MODO, BOBW, WEKI, WEME | 2 | HOLA, WEME |
| File-46.wav | 4 | GRSP, HOLA, PHEA, WEME | 4 | DICK, GRSP, PHEA, WEME |
| File-47.wav | 3 | GRSP, PHEA, WEME | 5 | DICK, GRSP, MODO, PHEA, WEME |
| File-48.wav | 3 | GRSP, PHEA, WEME | 6 | DICK, GRSP, MODO, PHEA, RWBL, WEME |
| File-49.wav | 0 | NONE | 5 | DICK, GRSP, PHEA, RWBL, WEME |
| File-50.wav | 5 | DICK, GRSP, HOLA, PHEA, WEME | 5 | DICK, GRSP, MODO, PHEA, WEME |

^1^ Species Codes: BARN = Barn Swallow, BLGR = Blue Grosbeak, BLUE = Blue Jay, BOBW = Bobwhite, CONI = Common Nighthawk, DICK = Dickcissel, EAKI = Eastern Kingbird, GOLD = American Goldfinch, GRAC = Common Grackle, GRSP = Grasshopper Sparrow, HOLA = Horned Lark, MODO = Mourning Dove, PHEA = Ring-necked Pheasant, ROBI = American Robin, RWBL = Red-winged Blackbird, SASP = Savannah Sparrow, TRSW = Tree Swallow, TURK = Wild Turkey, WEKI = Western Kingbird, WEME = Western Meadowlark

**S3 Table. Summary table of all bird species detected by BirdNET during the study period.**

| **Common Name** | **N Detections** | **Max C** | **Confirmed (Y/N)** |  | **Common Name** | **N Detections** | **Max C** | **Confirmed (Y/N)** |
| --- | --- | --- | --- | --- | --- | --- | --- | --- |
| **American Goldfinch** | **4,004** | **1.000** | **Y** |  | **Horned Lark** | **581,287** | **1.000** | **Y** |
| **American Robin** | **75,153** | **1.000** | **Y** |  | **Mourning Dove** | **31,117** | **1.000** | **Y** |
| **Barn Swallow** | **9,045** | **1.000** | **Y** |  | **Northern Bobwhite** | **34,607** | **1.000** | **Y** |
| **Blue Grosbeak** | **26,670** | **1.000** | **Y** |  | **Red-winged Blackbird** | **148,572** | **0.997** | **Y** |
| **Blue Jay** | **6,539** | **0.998** | **Y** |  | **Ring-necked Pheasant** | **13,151** | **1.000** | **Y** |
| **Common Grackle** | **8,407** | **1.000** | **Y** |  | **Savannah Sparrow** | **4,739** | **0.998** | **Y** |
| **Common Nighthawk** | **26,179** | **1.000** | **Y** |  | **Tree Swallow** | **7,637** | **0.996** | **Y** |
| **Dickcissel** | **397,405** | **1.000** | **Y** |  | **Western Kingbird** | **4,958** | **1.000** | **Y** |
| **Eastern Kingbird** | **28,748** | **1.000** | **Y** |  | **Western Meadowlark** | **1,331,498** | **1.000** | **Y** |
| **Grasshopper Sparrow** | **956,111** | **1.000** | **Y** |  | **Wild Turkey** | **4,083** | **1.000** | **Y** |
| American Avocet | 369 | 0.990 | N |  | House Wren | 273 | 0.958 | Y |
| American Crow | 495 | 0.997 | Y |  | Indigo Bunting | 192 | 0.811 | Y |
| American Kestrel | 968 | 1.000 | Y |  | Killdeer | 32,573 | 1.000 | Y |
| American Pipit | 922 | 0.999 | Y |  | Lark Bunting | 392,866 | 1.000 | Y |
| American Wigeon | 443 | 0.998 | N |  | Lark Sparrow | 3,659 | 1.000 | Y |
| American Tree Sparrow | 1,431 | 1.000 | Y |  | Lesser Black-backed Gull | 8 | 0.541 | N |
| Baird's Sandpiper | 1,563 | 0.897 | N |  | Least Sandpiper | 1,261 | 0.989 | Y |
| Bald Eagle | 186 | 0.943 | N |  | Little Blue Heron | 141 | 0.414 | N |
| Baltimore Oriole | 804 | 0.977 | Y |  | Lincoln's Sparrow | 104 | 0.923 | Y |
| Belted Kingfisher | 18,058 | 1.000 | Y |  | Long-billed Dowitcher | 2,051 | 0.998 | Y |
| Bell's Vireo | 968 | 0.998 | Y |  | Loggerhead Shrike | 2,308 | 1.000 | Y |
| Black-capped Chickadee | 710 | 0.983 | Y |  | Mallard | 935 | 0.998 | Y |
| Black-headed Grosbeak | 668 | 0.910 | N |  | Nashville Warbler | 14 | 0.491 | N |
| Black Tern | 321 | 0.985 | N |  | Northern Cardinal | 341 | 0.838 | Y |
| Brown-headed Cowbird | 8,445 | 1.000 | Y |  | Northern Flicker | 1,483 | 0.993 | Y |
| Bonaparte's Gull | 204 | 0.620 | N |  | Northern Harrier | 4,105 | 0.976 | Y |
| Brewer's Blackbird | 13 | 0.945 | N |  | Northern Mockingbird | 196 | 0.814 | Y |
| Barn Owl | 1,539 | 0.998 | Y |  | Northern Pintail | 823 | 0.997 | Y |
| Brown Thrasher | 3,183 | 0.999 | Y |  | Northern Shoveler | 1,207 | 0.995 | Y |
| Bufflehead | 18,377 | 0.901 | N |  | Northern Rough-winged Swallow | 387 | 1.000 | Y |
| Blue-gray Gnatcatcher | 13 | 0.804 | N |  | Orchard Oriole | 8,780 | 1.000 | Y |
| Blue-winged Teal | 11,497 | 1.000 | N |  | Orange-crowned Warbler | 39 | 0.918 | N |
| California Gull | 8 | 0.454 | N |  | Osprey | 796 | 0.994 | Y |
| Canada Goose | 8,935 | 0.996 | Y |  | Pied-billed Grebe | 1,975 | 0.998 | Y |
| Cassin's Sparrow | 5,958 | 0.831 | N |  | Red-bellied Woodpecker | 323 | 0.999 | Y |
| Cattle Egret | 208 | 0.968 | N |  | Redhead | 56 | 0.917 | N |
| Cedar Waxwing | 2,013 | 0.999 | Y |  | Red-eyed Vireo | 36 | 0.428 | N |
| Chipping Sparrow | 708 | 0.911 | N |  | Red-headed Woodpecker | 4,396 | 1.000 | Y |
| Chimney Swift | 471 | 1.000 | Y |  | Red-tailed Hawk | 948 | 0.998 | Y |
| Clay-colored Sparrow | 41,759 | 1.000 | N |  | Ring-billed Gull | 804 | 0.843 | N |
| Cliff Swallow | 712 | 1.000 | Y |  | Ring-necked Duck | 6,604 | 0.968 | N |
| Common Goldeneye | 32 | 0.881 | N |  | Rock Wren | 362 | 0.992 | Y |
| Common Merganser | 61 | 0.955 | N |  | Rough-legged Hawk | 211 | 0.998 | N |
| Common Tern | 147 | 0.891 | N |  | Ruby-crowned Kinglet | 10 | 0.844 | N |
| Common Yellowthroat | 2,040 | 0.976 | Y |  | Ruddy Duck | 516 | 0.873 | N |
| Cooper's Hawk | 54 | 0.586 | N |  | Sandhill Crane | 523 | 1.000 | Y |
| Dark-eyed Junco | 157 | 0.722 | N |  | Sanderling | 1,805 | 0.875 | Y |
| Double-crested Cormorant | 144 | 0.581 | N |  | Say's Phoebe | 1,171 | 1.000 | Y |
| Downy Woodpecker | 677 | 0.981 | Y |  | Short-eared Owl | 60 | 0.932 | N |
| Eared Grebe | 19 | 0.481 | N |  | Sharp-shinned Hawk | 94 | 0.956 | N |
| Eastern Bluebird | 2,036 | 0.998 | Y |  | Snow Goose | 525 | 0.999 | Y |
| Eastern Phoebe | 45 | 0.994 | Y |  | Solitary Sandpiper | 1,019 | 1.000 | Y |
| Eurasian Collared-Dove | 5,100 | 1.000 | Y |  | Song Sparrow | 817 | 0.993 | Y |
| European Starling | 9,090 | 0.999 | Y |  | Sora | 122 | 0.955 | N |
| Field Sparrow | 2,953 | 0.866 | Y |  | Spotted Sandpiper | 922 | 0.980 | Y |
| Forster's Tern | 45 | 0.472 | N |  | Spotted Towhee | 16 | 0.437 | Y |
| Franklin's Gull | 204 | 0.998 | Y |  | Swainson's Hawk | 527 | 0.978 | N |
| Gadwall | 426 | 1.000 | Y |  | Townsend's Solitaire | 211 | 0.989 | N |
| Green-winged Teal | 3,662 | 0.999 | Y |  | Upland Sandpiper | 33,004 | 1.000 | Y |
| Great Blue Heron | 2,215 | 0.999 | Y |  | Vesper Sparrow | 104,217 | 1.000 | Y |
| Great Crested Flycatcher | 639 | 0.998 | Y |  | Warbling Vireo | 83 | 0.906 | Y |
| Great Egret | 1,390 | 0.966 | N |  | Western Grebe | 3,947 | 0.999 | N |
| Greater Scaup | 94 | 0.426 | N |  | Western Wood-Pewee | 8 | 0.534 | N |
| Greater Yellowlegs | 919 | 0.999 | Y |  | White-breasted Nuthatch | 549 | 0.991 | Y |
| Great Horned Owl | 25,491 | 1.000 | Y |  | White-crowned Sparrow | 847 | 0.980 | Y |
| Greater Prairie-Chicken | 57,417 | 1.000 | Y |  | White-winged Dove | 497 | 0.846 | N |
| Gray Catbird | 116 | 0.868 | Y |  | Wilson's Warbler | 39 | 0.680 | N |
| Hairy Woodpecker | 221 | 0.996 | Y |  | Wood Duck | 170 | 1.000 | Y |
| Harris's Sparrow | 128 | 0.511 | N |  | American Coot | 1,646 | 0.957 | N |
| Herring Gull | 201 | 0.803 | Y |  | Yellow-breasted Chat | 1,474 | 0.988 | N |
| Hooded Merganser | 68 | 0.946 | N |  | Yellow-billed Cuckoo | 285 | 0.965 | N |
| Horned Grebe | 134 | 0.952 | N |  | Yellow-headed Blackbird | 34,123 | 1.000 | Y |
| House Finch | 1,663 | 0.998 | Y |  | Yellow Warbler | 35 | 0.693 | Y |
| House Sparrow | 559 | 0.954 | Y |  | Yellow-rumped Warbler | 8 | 0.427 | N |

**S1 Fig. Acoustic Monitor attached to the top of a polyvinyl chloride (PVC) pole.**

**
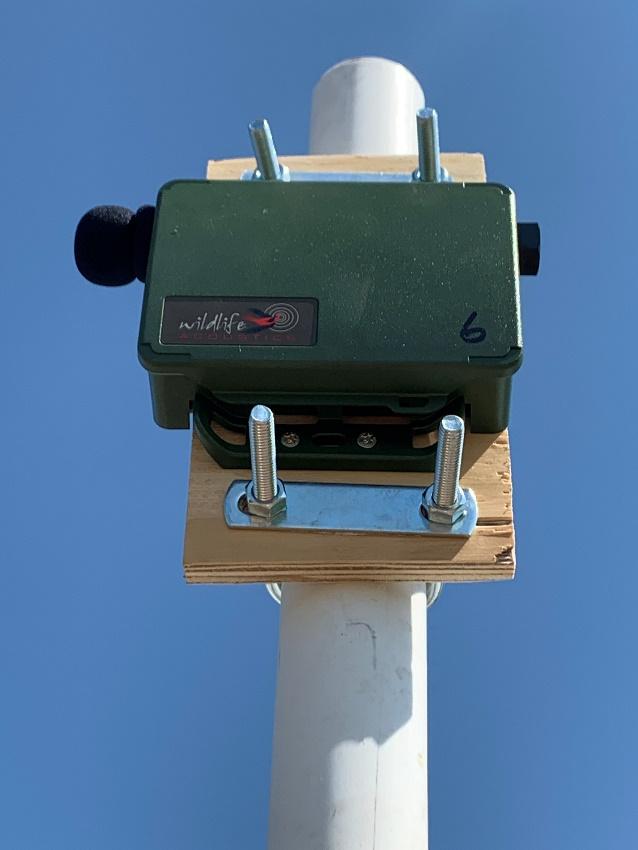
**

**S2 Fig. Acoustic recorder installed in the center pivot area of a corn field.**

**
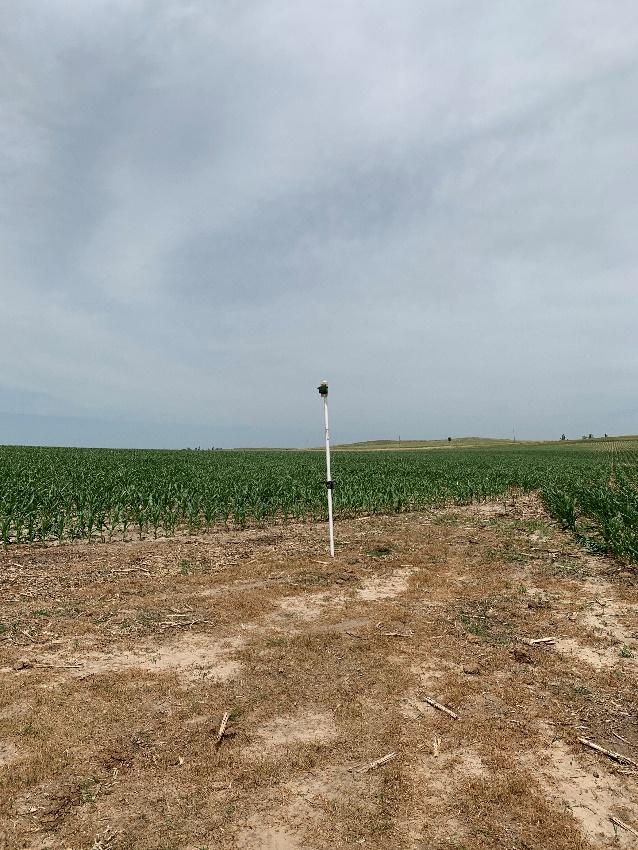
**

**S3 Fig. Acoustic recorder installed in a grassland field.**

**
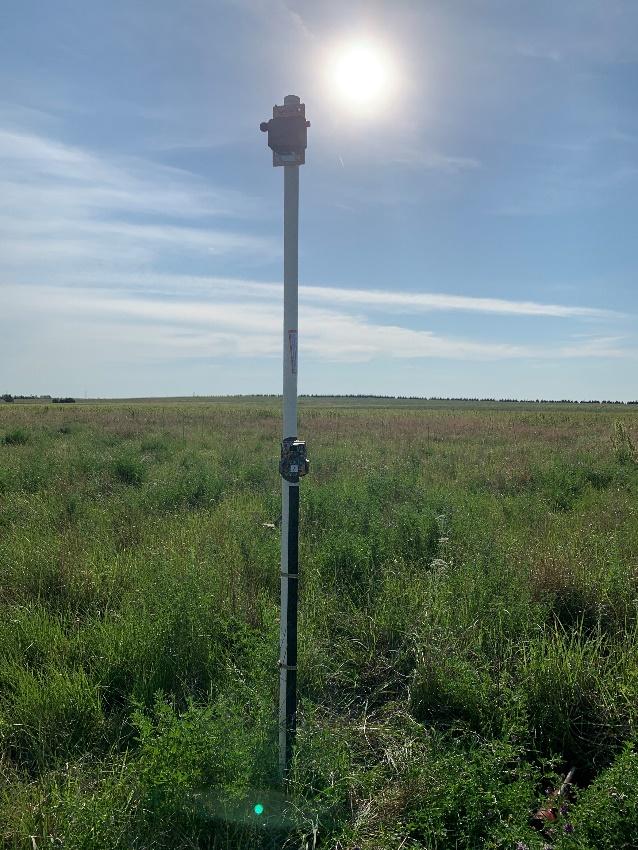
**
