# Supplementary material for: Evaluation of an autonomous acoustic surveying technique for grassland bird communities in Nebraska
Source: PLoS One. 2024 Jul 5;19(7):e0306580. doi: 10.1371/journal.pone.0306580 (PMC11226020; doi:10.1371/journal.pone.0306580)
Supplement: Attachment — Submitted filename: Response to reviewers.docx [file pone.0306580.s002.docx]

**Responses to Academic Editor Comments**

Academic Editor Comment: “Please ensure that your manuscript meets PLOS ONE's style requirements, including those for file naming. The PLOS ONE style templates can be found at
https://journals.plos.org/plosone/s/file?id=wjVg/PLOSOne_formatting_sample_main_body.pdf and
<https://journals.plos.org/plosone/s/file?id=ba62/PLOSOne_formatting_sample_title_authors_affiliations.pdf>”

**Author’s Response:** We formatted the revised manuscript to the best of our abilities to conform to the journal style requirements.

Academic Editor Comment: “Thank you for stating the following in the Acknowledgments Section of your manuscript: "Funding was provided by the US DOE Office of Energy Efficiency and Renewable Energy (EERE) Bioenergy Technologies Office under award DE-EE0009279. The views expressed in the article do not necessarily represent the views of the DOE or the US Government. The U.S. Government retains for itself, and others acting on its behalf, a paid-up nonexclusive, irrevocable worldwide license in said article to reproduce, prepare derivative works, distribute copies to the public, and perform publicly and display publicly, by or on behalf of the Government. We are grateful to Ryan Lamont, Hunter Swanson, and Wyatt Ervin for field assistance. We are also appreciative of the many private landowners that permitted site access for this study. Finally, we greatly appreciate the two anonymous reviewers and the associate editor that took time to provide feedback on earlier drafts."”

We note that you have provided funding information that is currently declared in your Funding Statement. However, funding information should not appear in the Acknowledgments section or other areas of your manuscript. We will only publish funding information present in the Funding Statement section of the online submission form.
Please remove any funding-related text from the manuscript and let us know how you would like to update your Funding Statement. Currently, your Funding Statement reads as follows:
"GS, LW, and AL received a Department of Energy grant to fund this work. The specific grant number is DE-EE0009279. No commercial companies funded this study or any of the authors. This is the URL to the funder website: https://www.energy.gov/eere/bioenergy/bioenergy-technologies-office

The funders did not play a role in study design, data collection and analysis, decision to publish, or preparation of the manuscript. "

Please include your amended statements within your cover letter; we will change the online submission form on your behalf.

**Author’s Response:** We removed the portion of the Acknowledgement section that described funding information and we included the updated funding statement within the cover letter.

Academic Editor Comment: “We note that Figure 1 in your submission contain map/satellite images which may be copyrighted. All PLOS content is published under the Creative Commons Attribution License (CC BY 4.0), which means that the manuscript, images, and Supporting Information files will be freely available online, and any third party is permitted to access, download, copy, distribute, and use these materials in any way, even commercially, with proper attribution. For these reasons, we cannot publish previously copyrighted maps or satellite images created using proprietary data, such as Google software (Google Maps, Street View, and Earth). For more information, see our copyright guidelines: http://journals.plos.org/plosone/s/licenses-and-copyright.

We require you to either (1) present written permission from the copyright holder to publish these figures specifically under the CC BY 4.0 license, or (2) remove the figures from your submission:

a. You may seek permission from the original copyright holder of Figure 1 to publish the content specifically under the CC BY 4.0 license.

We recommend that you contact the original copyright holder with the Content Permission Form (http://journals.plos.org/plosone/s/file?id=7c09/content-permission-form.pdf) and the following text:
“I request permission for the open-access journal PLOS ONE to publish XXX under the Creative Commons Attribution License (CCAL) CC BY 4.0 (http://creativecommons.org/licenses/by/4.0/). Please be aware that this license allows unrestricted use and distribution, even commercially, by third parties. Please reply and provide explicit written permission to publish XXX under a CC BY license and complete the attached form.”

Please upload the completed Content Permission Form or other proof of granted permissions as an ""Other"" file with your submission.

In the figure caption of the copyrighted figure, please include the following text: “Reprinted from [ref] under a CC BY license, with permission from [name of publisher], original copyright [original copyright year].”

b. If you are unable to obtain permission from the original copyright holder to publish these figures under the CC BY 4.0 license or if the copyright holder’s requirements are incompatible with the CC BY 4.0 license, please either i) remove the figure or ii) supply a replacement figure that complies with the CC BY 4.0 license. Please check copyright information on all replacement figures and update the figure caption with source information. If applicable, please specify in the figure caption text when a figure is similar but not identical to the original image and is therefore for illustrative purposes only.
The following resources for replacing copyrighted map figures may be helpful:

USGS National Map Viewer (public domain): http://viewer.nationalmap.gov/viewer/
The Gateway to Astronaut Photography of Earth (public domain): http://eol.jsc.nasa.gov/sseop/clickmap/
Maps at the CIA (public domain): https://www.cia.gov/library/publications/the-world-factbook/index.html and https://www.cia.gov/library/publications/cia-maps-publications/index.html
NASA Earth Observatory (public domain): http://earthobservatory.nasa.gov/
Landsat: http://landsat.visibleearth.nasa.gov/
USGS EROS (Earth Resources Observatory and Science (EROS) Center) (public domain): http://eros.usgs.gov/#
Natural Earth (public domain): <http://www.naturalearthdata.com/>”

**Author’s Response:** Figure 1 was produced using ESRI World Imagery in ArcGIS Pro. Based on ESRI’s Terms of Use (e.g., <https://doc.arcgis.com/en/arcgis-online/reference/static-maps.htm>), we have permission to use static maps of ESRI World Imagery in academic publications such as this. We have updated the figure to credit ESRI for the source imagery. In addition, we confirmed that the use of the Basemap aligns with the conditions of the Creative Common Attribution 4.0 International license by reaching out to ESRI and obtaining a copyright permission letter which is attached to the revised submission.

Academic Editor Comment: “Please review your reference list to ensure that it is complete and correct. If you have cited papers that have been retracted, please include the rationale for doing so in the manuscript text, or remove these references and replace them with relevant current references. Any changes to the reference list should be mentioned in the rebuttal letter that accompanies your revised manuscript. If you need to cite a retracted article, indicate the article’s retracted status in the References list and also include a citation and full reference for the retraction notice.”

**Author’s Response:** We reviewed the reference list to ensure that all references are in the correct format. Three references were added to the reference list [13, 14, 27] and the order of the references was changed, but no references were removed.

**Responses to Reviewer #1 Comments**

Reviewer 1 General Comment: “This is a very interesting, well written manuscript. It is very clear, aims are nicely defined, methods are appropriate, and results reflect aims. Nonetheless, I found some minor issues, listed below, to be addressed before publishing.”

**Author's Response:** We greatly appreciate the time you took to review our manuscript and provide feedback.

Reviewer 1 Comment: “Lines 105-106: as I guess, did you monitor breeding birds? If yes, please, add this detail because could be wintering birds.”

**Author’s Response:** We focused on grassland birds during the breeding season, but the avian species included in this study consisted of both breeding grassland birds as well as year-round residents of Nebraska. This detail was added to the manuscript to enhance clarity.

Reviewer 1 Comment: “Line 124: replace “acoustic monitors” with “ARUs””

**Author’s Response:** We abbreviated ‘acoustic monitors’ to ‘ARUs’.

Reviewer 1 Comment: “Lines 128-129: did you placed ARUs in all these grassland types? Or only in one of them? Please specified.”

**Author’s Response:** We did not specifically place ARU’s in these grassland types, they were just the dominant land types of this region. Instead we placed the monitors in Conservation Reserve Program (CRP) sites and to improve clarity, we did include the dominant grass species that were present at each of our grassland study sites.

Reviewer 1 Comment: “Line 142: remove “separate””

**Author’s Response:** The word ‘separate’ was removed.

Reviewer 1 Comment: “Did you used some detection radius to count birds or did you count birds with “unlimited” distance (so, all birds seen/heard also at great distance)?”

**Author’s Response:** We are assuming that this comment is referring to our point count methods. During our point counts, we did not have a specific detection radius to count birds, mostly because the landscape is so open and flat you can hear birds from great distances with confidence. We did specify the lack of detection radius within our methods.

Reviewer 1 Comment: “Line 156: replace “acoustic monitors” with “ARUs”.”

**Author’s Response:** We abbreviated ‘acoustic monitors’ to ‘ARUs’.

Reviewer 1 Comment: “Line 158: replace “space” with “distance””

**Author’s Response:** We replaced the word ‘space’ with ‘distance’.

Reviewer 1 Comment: “Lines 162-163: why? To avoid the edge effect? If yes, please add this fact to be more clear.”

**Author’s Response:** We made sure that the acoustic monitors were at least 100 meters away from the field edge not necessarily to avoid edge effect, but rather to make sure that the bird species detected was within our study site and not occupying a neighboring field. We added this detail to make the methods more clear.

Reviewer 1 Comment: “Line 272: replace “to comparing” with “to compare””

**Author’s Response:** If this comment is referring to the sentence “In addition to comparing focal species richness, we compared total species richness estimates over the 60-day point count survey period” we feel that “to comparing” is the correct expression.

Reviewer 1 Comment: “Lines 391 and 393: replace Fig. A and Fig. B with Panel A and Panel B.”

**Author’s Response:** We replaced ‘Fig. A’ and ‘Fig. B’ with ‘Panel A’ and ‘Panel B’ in the revised manuscript.

**Responses to Reviewer #2 Comments**

Reviewer 2 General Comment: “I have read the study with pleasure. It is a really good and valuable paper examining the effectiveness of a completely automatic acoustic approach to bird monitoring. The authors proved that by applying soundscape recording and automatic detection of bird vocalizations using BirdNet, they achieve the same or better level of effectiveness as human observers. They have really well-planned the study, estimated the recall and precision of automatic detections, and manually confirmed the detection of each species by BirdNet. In my opinion, the study should be published after minor revision.”

**Author’s Response:** We greatly appreciate the time you took to review our manuscript and provide feedback.

Reviewer 2 Comment: “L56-63: You should also point out some limitations. The first one is that PAM focuses only on vocalizing animals, thus we assume that vocalizing animals reflect overall biodiversity. The next issue is unequal detection range for differently vocalizing species (depends on frequency and amplitude of vocalisation). Additionally, biodiversity is not all; species abundance is also important but difficult to catch by PAM.”

**Author’s Response:** Limitations surrounding the challenges with only focusing on vocalizing animals, estimating species abundance using PAMs, and the potential difficulties with detecting some species that are less vocal were added.

Reviewer 2 Comment: “L99-104: The BirdNet does not detect signals on its own but compares the time period (3 sec) with the template. Thus, we may expect that the effectiveness of bird detection by BirdNet may differ between different bird communities and may vary depending on the time of day or season. Therefore, it is important to test the effectiveness of BirdNet under various ecological conditions. Your study does this, which is why it is important.”

**Author’s Response:** Thank you for this comment. We agree**.**

Reviewer 2 Comment: “L123: Please provide more information about the timing of the breeding season within your study area. Additionally, could you share some basic information about species richness?”

**Author’s Response:** The timing of the grassland birds breeding season in our study area was added. Regarding species richness, we included a list of all confirmed species detections (ARUs and Point Counts) in the supplemental information.

Reviewer 2 Comment: “L159: Did you check the microphones before recording? Which gain setting did you apply?”

**Author’s Response:** The gain setting was left at the default setting (18 dB) and microphones were checked before installation. We specified this in the new manuscript draft.

Reviewer 2 Comment: “L222: Is it not too low sample size obtain a good estimation of recall? Especially for rare or low-vocalizing species, you will get no detections.”

**Author’s Response:** Fifty 120-second clips is an adequate sample size for estimating recall for our 20 common focal grassland bird species. Since we choose avian species that are highly vocal and make up the bulk of detections at each of our study sites, we felt it was likely we would detect each of these species within the timeframe. Unfortunately, due to choosing 120 random clips, we did not detect three focal species. While studies that are focusing on rare or low-vocalizing species should have a larger sample size, we feel the sample size we used allowed us to answer our research question. We made revisions in the discussion portion of the manuscript to clarify these shortcomings of calculating recall from low sample sizes.
